# Identification of *PTPN22* as a potential genetic biomarker for abdominal aortic aneurysm

**DOI:** 10.3389/fcvm.2022.1061771

**Published:** 2022-12-14

**Authors:** Peng Ruan, Lei Gao, Hui Jiang, Tianshu Chu, Jianjun Ge, Xiang Kong

**Affiliations:** Department of Cardiovascular Surgery, The First Affiliated Hospital of USTC, Division of Life Sciences and Medicine, University of Science and Technology of China (USTC), Hefei, Anhui, China

**Keywords:** abdominal aortic aneurysm, diagnosis, biomarker, *PTPN22*, cellular AAA model

## Abstract

Abdominal aortic aneurysm (AAA) is a severe life-threatening disease that is generally asymptomatic and is diagnosed at a very late stage. The genetic component underpinning AAA is considerable, with an estimated heritability of up to 70%. Therefore, identifying genetic biomarkers for AAA is valuable for predicting high-risk populations. We used integrative bioinformatics and cellular AAA model-based validation to reveal that the gene encoding protein tyrosine phosphatase non-receptor type 22 (*PTPN22*) may be a potentially useful diagnostic biomarker for AAA. Integrative bioinformatics analyses of clinical specimens showed that *PTPN22* expression was consistently upregulated in aortic tissues and peripheral blood mononuclear cells (PBMCs) derived from patients with AAA. Moreover, transcriptomics data revealed that *PTPN22* is a potential biomarker for AAA with limited diagnostic value in patients with thoracic aortic aneurysm/dissection. Single-cell RNA sequencing-based findings further highlight *PTPN22* expression in aortic immune cells and vascular smooth muscle cells (VSMCs) is consistently upregulated in patients with AAA. A cellular AAA model was eventually employed to verify the increase in *PTPN22* expression. Collectively, the results indicate that *PTPN22* could be a potentially useful diagnostic biomarker for AAA.

## Introduction

Abdominal aortic aneurysm (AAA) is a severe life-threatening disease with an overall incidence of 6% in men and 1.6% in women (1). The hallmark of AAA is localized enlargement of the infrarenal aorta with a diameter of > 3.0 cm (2). Unless there is a rapid increase in size or rupture, AAA is generally asymptomatic and is diagnosed at a very late stage (1). The occurrence of AAA is highly associated with an unhealthy lifestyle, such as a history of smoking or hypertension (3). However, the genetic component underpinning AAA is substantial, with an estimated heritability of up to 70% (4). Difficulties remain in terms of the implementation of screening programs, and current knowledge on the genetic component of AAA is insufficient to guide early screening in the clinic. Therefore, identifying biomarkers for AAA is valuable for predicting high-risk populations (5).

High-throughput platform-based biomarker identification has been highlighted as a promising approach for the diagnosis and prevention of AAA and other diseases (6–8). The discovery of therapeutic targets is generally based on a consistent increase or decrease in the expression of target genes in patients and/or experimental models, followed by genetic manipulation and/or targeted drug-based screening, which allow confirmation of the therapeutic targets and subsequent identification of feasible treatment options (9–12). Integrative bioinformatics and experimental validation are highly efficient tools for identifying biomarkers to accurately predict the occurrence of AAA (6, 13). As proof-of-concept, several publications have employed these research strategies to identify biomarkers with diagnostic and prognostic value in several diseases (7, 8, 14–16).

In the current study, we used a comprehensive bioinformatics-based analysis and experimental validation to verify the gene encoding protein tyrosine phosphatase non-receptor type 22 (*PTPN22*) as a potentially specific biomarker for patients with AAA.

## Results

### Identification of *PTPN22*, *CPVL*, *ARHGDIB*, and *ANGPTL6* as potential biomarkers for patients with AAA

**FIGURE 1:**
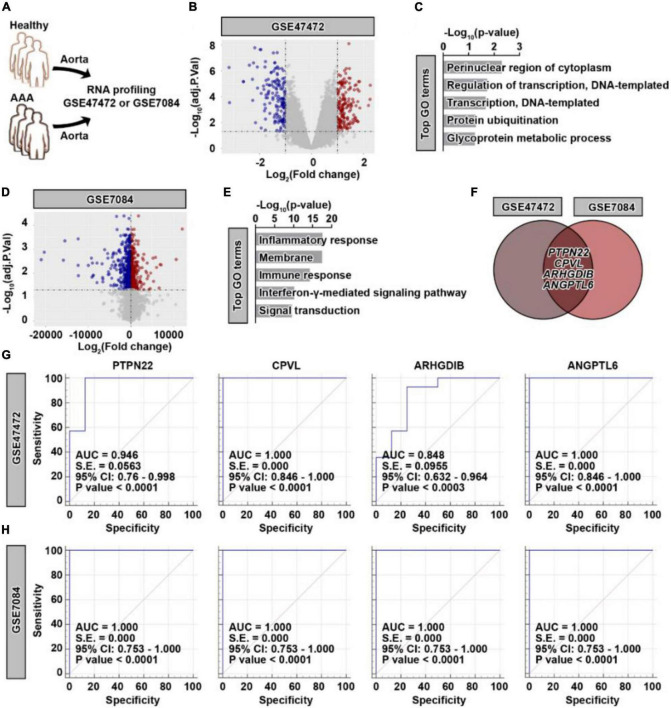
Identification of four potential genetic biomarkers for abdominal aortic aneurysm (AAA). **(A)** Schematic of the GSE47472 and GSE7084 datasets. **(B)** Volcano plot of differentially expressed genes (DEGs) in AAA samples based on the GSE47472 dataset. Red, significantly upregulated genes; blue, significantly downregulated genes; gray, no significant difference. A fold change of ≥ 2 and a *p-*value of < 0.05 were considered statistically significant. **(C)** The top enriched Gene Ontology (GO) terms of significantly upregulated DEGs in AAA samples. **(D)** Volcano plot of DEGs in AAA samples based on the GSE7084 dataset. Red, significantly upregulated genes; blue, significantly downregulated genes; gray, no significant difference. A fold change of ≥ 2 and a *p-*value of < 0.05 were considered statistically significant. **(E)** The top enriched GO terms of significantly upregulated DEGs in AAA samples. **(F)** Venn diagram of significantly upregulated DEGs between the GSE47472 and GSE7084 datasets. Receiver operating characteristic curve analysis of *PTPN22*, *CPVL*, *ARHGDIB*, and *ANGPTL6* based on the GSE47472 **(G)** and GSE7084 **(H)** datasets.

To profile the genetic alterations in aortic tissues collected from patients with AAA, two datasets with similar grouping characteristics were retrieved (Figure 1A). As shown in Figure 1B, differentially expressed genes (DEGs) were identified in patients with AAA based on the GSE47472 dataset (Supplementary Figure 1). Among these DEGs, 169 were significantly upregulated, while 155 were significantly downregulated. The upregulated DEGs were highly enriched in biological processes, such as the perinuclear region of the cytoplasm and regulation of transcription (Figure 1C). Based on the GSE7084 dataset (Supplementary Figure 2), 1,653 AAA-specific DEGs were identified (Figure 1D), which consisted of 698 significantly upregulated and 955 significantly downregulated genes. Different from the GSE47472 dataset, these upregulated DEGs were highly enriched in the inflammatory response and the immune response (Figure 1E). To obtain more reliable candidates associated with AAA, we cross-compared the upregulated genes from both datasets. Four genes, including *PTPN22*, *CPVL*, *ARHGDIB*, and *ANGPTL6*, were concurrently upregulated (Figure 1F). The receiver operating characteristic (ROC) curve results based on the GSE47472 (Figure 1G) and GSE7084 (Figure 1H) datasets further revealed that these four candidates were highly predictive of the AAA diagnosis. These results suggest that *PTPN22*, *CPVL*, *ARHGDIB*, and *ANGPTL6* are potential diagnostic biomarkers for patients with AAA.

### *PTPN22* is a genetic biomarker for patients with AAA

**FIGURE 2:**
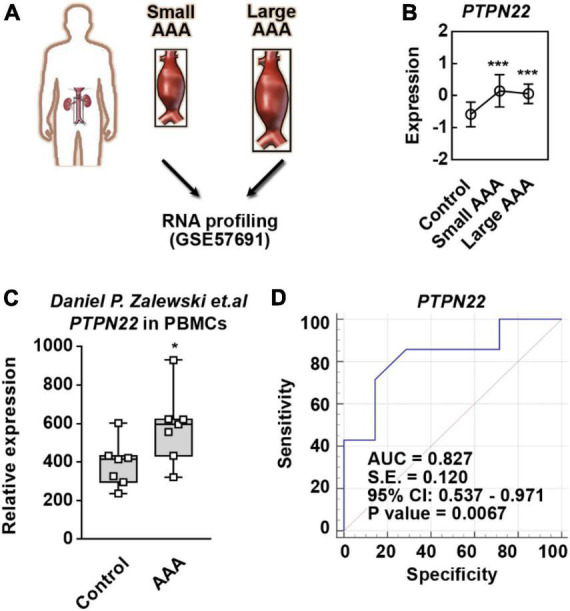
*PTPN22* is a genetic biomarker for abdominal aortic aneurysm (AAA). **(A)** Schematic of the GSE57691 dataset. Abdominal aortic tissues collected from patients with small and large AAA were profiled by RNA sequencing. Small AAA, mean maximum aortic diameter of 54.3 ± 2.3 mm; large AAA, mean maximum aortic diameter of 68.4 ± 14.3 mm. **(B)** *PTPN22* expression based on the GSE57691 dataset. The data are shown as the mean ± standard deviation (SD) of at least 10 patients. * Significantly different from control; ***p < 0.001. The expression **(C)** and receiver operating characteristic curve **(D)** analyses of *PTPN22* in peripheral blood mononuclear cell (PBMC) samples collected from control participants and patients with AAA based on an independent cohort. The data are shown as the mean ± SD of seven patients. *Significantly different from control; ***p < 0.001.

Considering that the aortic diameter in AAA is highly associated with the expression of genetic biomarkers, the GSE57691 dataset was analyzed to precisely identify reliable biomarkers (Figure 2A and Supplementary Figure 3). Among the four identified genes (*PTPN22*, *CPVL*, *ARHGDIB*, and *ANGPTL6*), we found that *PTPN22* expression was consistently upregulated in both small and large AAA samples (Figure 2B). To explore whether the increase in *PTPN22* expression is conserved among species, the GSE109039 dataset was retrieved (Supplementary Figures 4A,B). An exclusive increase in the expression of *PTPN22* was observed in the experimental AAA model (Supplementary Figure 4C). To further validate the expression and diagnostic value of *PTPN22*, an independent cohort of peripheral blood mononuclear cells (PBMCs) from patients with AAA was derived (17). As expected, *PTPN22* expression was significantly increased in PBMCs from AAA patients (Figure 2C). The diagnostic value (*p* = 0.0067) of *PTPN22* in patients with AAA was also confirmed by the ROC curve analysis (Figure 2D). Taken together, these results reveal that *PTPN22* is a genetic biomarker for patients with AAA.

### *PTPN22* expression in aortic immune cells is upregulated in patients with AAA

**FIGURE 3:**
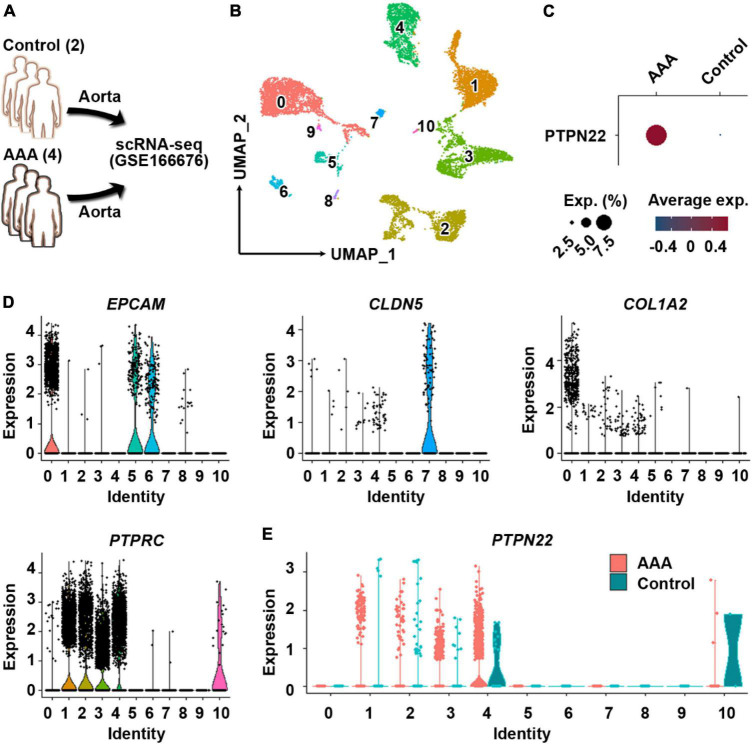
*PTPN22* upregulation in aortic tissues from patients with abdominal aortic aneurysm (AAA) is largely distributed in resident immune cells. **(A)** Schematic of the GSE166676 dataset. scRNA-seq, single-cell RNA sequencing. **(B)** Uniform manifold approximation and projection (UMAP) plot showing that aortic tissues were divided into 11 distinct clusters. **(C)** Dot plot showing that *PTPN22* expression was highly increased in aortic tissues from patients with AAA. **(D)** Violin plots showing the expression of *EPCAM*, *CLDN5*, *COL1A2*, and *PTPRC* among the 11 distinct clusters. **(E)** Violin plot showing the expression of *PTPN22* between control participants and patients with AAA among the 11 distinct clusters.

Single-cell RNA sequencing is a powerful tool that can be used to dissect cellular heterogeneity within tissues (18). To explore the potential cellular origin of upregulated *PTPN22* within aortic tissues from patients with AAA, the GSE166676 dataset, which is consisted of two healthy volunteers and four patients with AAA (18), was retrieved (Figure 3A). First, the clustering analysis revealed 10 distinct cell populations within aortic tissues (Figure 3B), which is consistent with a previous report (18). As shown in Figure 3C, *PTPN22* expression was consistently upregulated in aortic tissues from patients with AAA. To determine the potential origin, aortic cells were re-clustered using lineage-specific biomarkers, including *EPCAM* (epithelial cells), *CLDN5* (endothelial cells), *COL1A2* (mesenchymal cells), and *PTPRC* (immune cells) (Figure 3D). As shown in Figure 3E, the distribution of *PTPN22*-expressing cells was highly co-localized with clusters assigned as immune cells, suggesting that AAA-upregulated *PTPN22* may be derived from aortic immune cells. Collectively, these findings suggest that *PTPN22* expression in aortic immune cells is remarkably upregulated in patients with AAA.

### Dissecting the cellular distribution of upregulated *PTPN22* expression within aortic immune cells from patients with AAA

**FIGURE 4:**
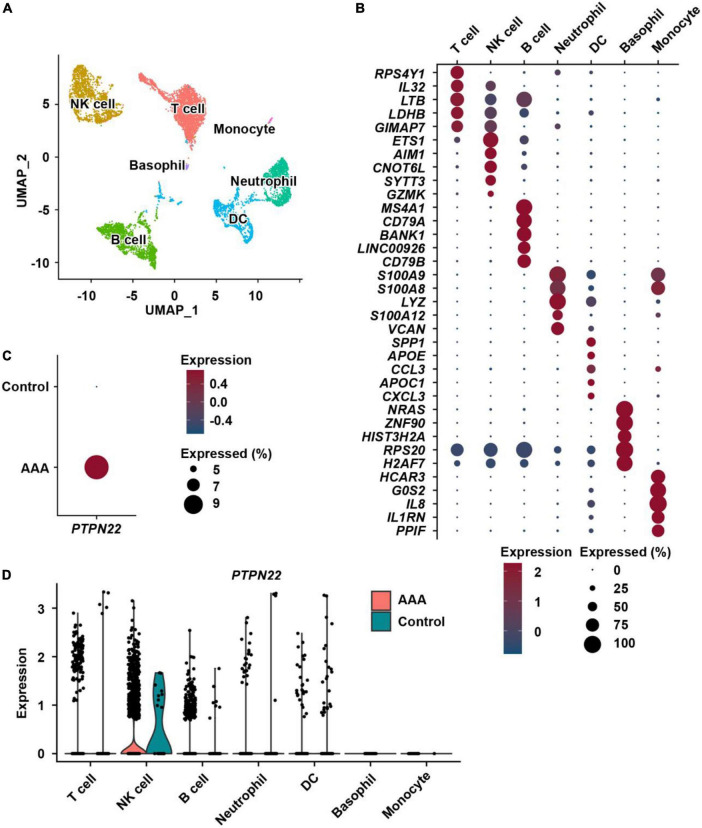
Distribution analysis of *PTPN22* within resident immune cells. **(A)** UMAP plot showing immune cell subpopulations within aortic tissues. NK cell, natural killer cell; DC, dendritic cell. **(B)** Dot plot of the top five marker genes for each immune cell subpopulation. **(C)** Dot plot showing that the expression of *PTPN22* was highly increased in immune cells from patients with AAA. **(D)** Violin plot showing the expression of *PTPN22* between control participants and patients with AAA among the distinct immune cell subpopulations.

To precisely determine the cellular origin of AAA-upregulated *PTPN22*, immune cells were extracted (Figure 4A) and re-clustered with their identities annotated using reported marker genes (Figure 4B). As expected, *PTPN22* expression in immune cells was also upregulated in AAA samples (Figure 4C) when compared with control samples. As shown in Figure 4D, *PTPN22* expression was uniquely distributed in aortic immune cell subpopulations, including T cells, natural killer (NK) cells, B cells, neutrophils, and dendritic cells (DCs), but not in basophils and monocytes. Together, these results reveal that upregulated *PTPN22* expression in immune cells from AAA patients is mainly distributed in the subpopulations, including T cells, NK cells, and B cells.

### *PTPN22* lacks diagnostic value for patients with thoracic aortic aneurysm/dissection (TAAD)

**FIGURE 5:**
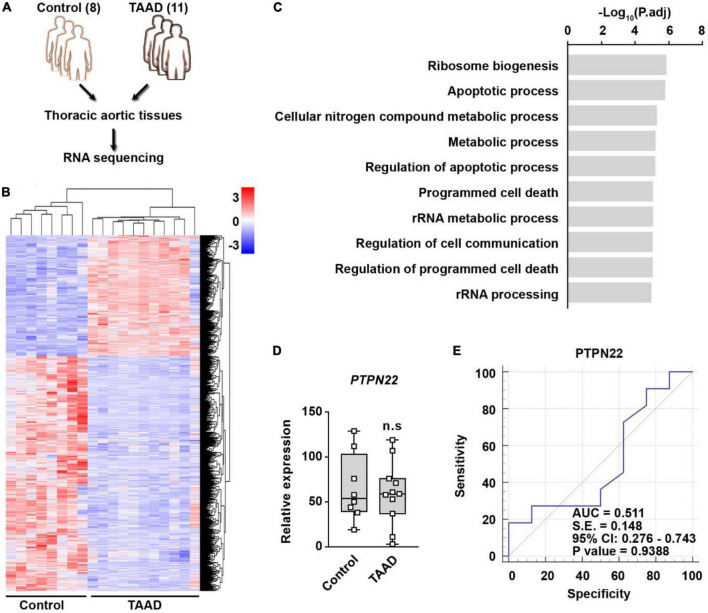
*PTPN22* lacks diagnostic value for TAAD. **(A)** Schematic of the experimental design. Thoracic aortic tissues collected from 11 patients with TAAD and 8 control participants were subjected to RNA sequencing. **(B)** Heatmap showing differentially expressed genes (DEGs) between control and TAAD samples. **(C)** The top enriched Gene Ontology terms of the significantly upregulated DEGs in TAAD samples. Expression **(D)** and receiver operating characteristic (ROC) curve **(E)** analyses of *PTPN22* in aortic tissues collected from control participants and patients with TAA. n.s., not significant.

To evaluate the expression and diagnostic value of *PTPN22* in patients with TAAD, thoracic aortic tissues from 11 patients with TAAD and 8 control participants were profiled by RNA sequencing (Figure 5A). We identified 909 DEGs that were significantly upregulated in TAAD samples, and the expression patterns of these genes among various samples are shown using heatmap (Figure 5B). The significantly upregulated DEGs were highly enriched in certain Gene Ontology (GO) terms, including programmed cell death and metabolic processes (Figure 5C), revealing the prominent role of cell death within aortic tissues during TAAD progression. However, unexpectedly, the expression of *PTPN22* in TAAD samples was unaltered when compared with control samples (Figure 5D). As shown in Figure 5E, the ROC curve showed that the expression of *PTPN22* lacked diagnostic value for TAAD in our aortic tissue-based analysis (*p* = 0.9388). Although TAAD and AAA are anatomically inextricable, they share several diversities (19, 20). The GSE9106 dataset, which included peripheral blood cell-based RNA profiling of TAA, was retrieved to evaluate the association of *PTPN22* with TAA (Supplementary Figures 5A,B). However, *PTPN22* expression was unaltered between controls and patients with TAA (Supplementary Figure 5C). The ROC curve analysis further showed that the area under the curve (AUC) for *PTPN22* (*p* = 0.6081) was 0.531 (Supplementary Figure 5D), indicating the limited diagnostic value of *PTPN22* for TAA. Collectively, these clinical TAAD sample-based findings confirm that the expression and diagnostic value of *PTPN22* is restricted to AAA, rather than TAAD.

### *PTPN22* expression is consistently upregulated in vascular smooth muscle cells (VSMCs) from AAA patients and cellular model

**FIGURE 6:**
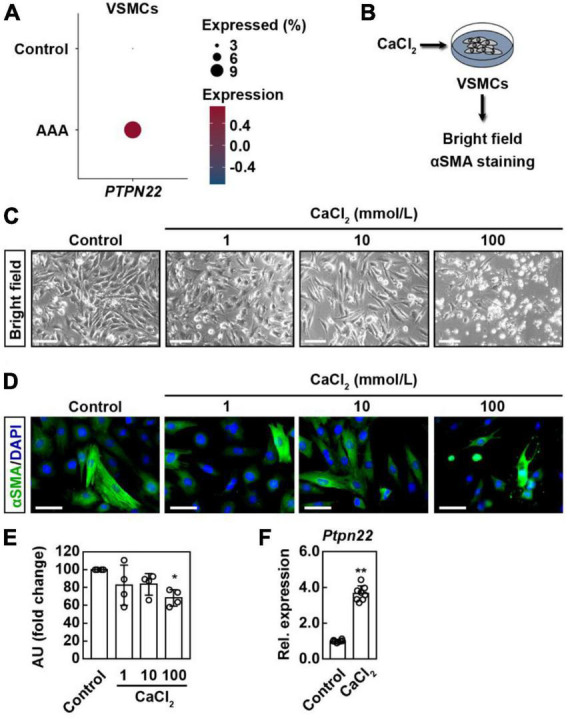
*PTPN22* expression is consistently upregulated in VSMCs from AAA patients and cellular model. **(A)** Dot plot showing that the expression of *PTPN22* was highly increased in vascular smooth muscle cells (VSMCs) from patients with AAA based on scRNA-seq data. **(B)** Schematic of the experimental design. Representative bright field **(C)** and immunofluorescence **(D)** images of VSMCs treated with different concentrations of calcium chloride (CaCl_2_). For **(C)**, representative images of four biological replicates from two independent experiments are shown. Scale bar, 100 μm. For **(D)**, the cells were stained with α-smooth muscle actin (α-SMA) (green), and the nuclei were labeled with DAPI (blue). Representative images of four biological replicates from two independent experiments are shown. Scale bar, 50 μm. For **(E)**, the data are shown as the mean ± standard deviation of four biological replicates. *Significantly different from control; *p < 0.05. AU, Arbitrary Unit. **(F)** VSMCs were treated with CaCl_2_ (100 mmol/L), and relative (Rel.) expression of cellular *PTPN22* was assessed by quantitative real-time polymerase chain reaction. The data are shown as the mean ± standard deviation of four biological replicates. *Significantly different from control; **p < 0.01.

VSMCs are the only cell type within the aortic media, and loss of VSMCs resulting from apoptotic cell death contributes etiologically to both human and experimental AAA (21, 22). To explore the potential role of *PTPN22* in aortic VSMCs, cells expressing *ACTA2*, a marker gene for VSMC, were further extracted from scRNA-seq data analyzed above (Supplementary Figure 6). As expected, the expression of *PTPN22* was consistently increased in VSMCs from AAA patients (Figure 6A). Although a murine AAA model induced by calcium chloride (CaCl_2_) has been well-established (23, 24), there is still a lack of ideal cellular AAA models for *in vitro* studies (25). To confirm that *PTPN22* expression was increased in the *in vitro* AAA model, a CaCl_2_-based apoptotic VSMC model was adopted (Figure 6B) (24, 26). As shown in Figure 6C, VSMCs were morphologically changed in response to CaCl_2_ treatment in a concentration-dependent manner. Consistent with a previous report (26), VSMCs treated with CaCl_2_ at a concentration of 100 mmol/L showed remarkably diminished α-smooth muscle actin (α-SMA) expression that did not extend entirely across the cell body (Figures 6D,E), suggesting that VSMCs treated with CaCl_2_ (100 mmol/L) can reproduce the aortic aneurysmal phenotype *in vitro*. Moreover, *PTPN22* expression was significantly increased in VSMCs treated with CaCl_2_, as assessed by quantitative real-time polymerase chain reaction (qRT-PCR) (Figure 6F). Taken together, these results further highlight the important role of *PTPN22* in AAA.

## Discussion

AAA is generally asymptomatic before rupture; thus, diagnosing AAA at an early stage and elucidating the underlying mechanisms leading to progressive dilatation are two major challenges in the clinic. Identification of genetic biomarkers is useful to develop novel diagnostic and therapeutic approaches (2). Previous studies have identified several potential biomarkers for AAA through studying the levels of different molecules related to the pathological mechanisms of AAA (27, 28). In this study, we demonstrated that *PTPN22* may be a valuable AAA-specific diagnostic biomarker. *PTPN22* was first associated with type 1 diabetes mellitus, and it has since demonstrated involvement in the pathogenesis of various autoimmune diseases, such as rheumatoid arthritis (29). Lymphoid tyrosine phosphatase, which is encoded by *PTPN22*, is expressed in all leukocyte linages. Although the increased expression and diagnostic value of *PTPN22* were verified using a peripheral blood sample-based cohort in this study, more PBMC-based examinations of *PTPN22* between healthy controls and patients with AAA need be conducted to confirm its clinical value as a biomarker for AAA. Moreover, *PTPN22* in circulating extracellular vesicles in peripheral blood also deserves to be investigated (30, 31).

People with high cholesterol and a history of smoking are at a high risk of AAA, and biomarker-based monitoring of patients diagnosed with AAA at an early stage also has long-term benefits. Thus, genetic screening among these populations is valuable (32). Two independent cohort-based genome-wide association studies for AAA have identified nine risk loci, and several genes have been reported to have vital functions in the pathogenesis of AAA (13, 33). Previous studies have also highlighted that targeting these crucial molecules could prevent AAA development (34, 35). For instance, imatinib can prevent aneurysm progression by inhibiting the expression and activation of matrix metallopeptidase 9 in experimental AAA models (35). Here, we identified that *PTPN22* may be a potential therapeutic target for AAA, which is supported by the observation that *PTPN22* expression is well replicated in experimental AAA models induced by CaCl_2_. Identification of therapeutic targets is generally based on a consistent upregulation or downregulation in the expression of target genes. Then, genetic manipulation and/or targeted drug-based screening allow the therapeutic targets to be confirmed, which could in turn help to identify feasible treatment options (9–12). Therefore, genetic deletion or small molecule-based inhibition of *PTPN22*, such as with PTPN22-IN-1, would be helpful for the development of therapeutic agents to prevent AAA progression (36).

AAA results from changes in the aortic wall structure, including thinning of the media and adventitia due to loss of VSMCs and extracellular matrix degradation (2). However, as a protein tyrosine phosphatase, the intracellular distribution of the protein encoded by *PTPN22* is largely restricted to the nucleus and cytoplasm. Here we provide the first evidence at single-cell level that a remarkable increase in the expression of *PTPN22* in both aortic immune cells and VSMCs from patients with AAA. However, functionally, the protein encoded by *PTPN22* serves as a intracellularly negative regulator of T cell receptor signaling. Therefore, it seems unlikely that this protein would be secreted from immune cells and VSMCs as its upregulated (37). To the best of our knowledge, no previous studies have determined whether the protein encoded by *PTPN22* can be secreted by aortic immune cells and/or VSMCs, as well as its intermediate role between these two type of cells within aortic tissues during the pathogenesis of AAA. We thus speculate that the steady state within local aortic tissues would be disrupted as a result of a remarkable increase in *PTPN22* expression in aortic immune cells and/or VSMCs. We believe that a cascade amplification reaction resulting from immune cells and/or VSMCs mediated by *PTPN22* upregulation may play a critical role in the occurrence and progression of AAA.

Although AAA and TAAD share multiple similarities, they also demonstrate several differences, including the population prevalence, mode of inheritance, and predisposing genes (19). Genetic disorders and congenital factors are common causes of TAAD. For example, mutations in the gene encoding fibrillin-1 can cause progressive ascending aortic dilatation. The aortic wall of the ascending aorta also has a higher blood pressure than the abdominal aorta (38). Atherosclerosis is the most important risk factor for AAA (19), and atherosclerotic tissues generally demonstrate excessive resident immune cell activation and aberrant activation of VSMCs (39, 40). This microenvironment may lead to a cascade amplification of PTPN22 production by immune cells and VSMCs, resulting in a significant increase in expression in local arterial immune cells and VSMCs in patients with AAA. The difference in the expression of *PTPN22* between patients with TAAD and patients with AAA may be largely due to differences in the mechanisms leading to these diseases. We thus believe that more in-depth basic and clinical research is needed to understand the difference in the expression of *PTPN22* between patients with AAA and those with TAAD.

In conclusion, our integrative bioinformatics analyses have highlighted the potential diagnostic and therapeutic value of *PTPN22* as a biomarker for AAA. However, more experimental and clinical studies should be conducted to further elucidate the importance of *PTPN22* in AAA.

## Materials and methods

### Data collection and processing

The datasets were retrieved from the Gene Expression Omnibus database^1^ and were analyzed using RStudio. An adjusted *p-*value of < 0.05 and a | Log2FC| of ≥ 1 were considered statistically significant. The GSE47472 dataset contained 8 control aortic tissues and 14 AAA neck tissues (41), and the GSE7084 dataset contained 8 control abdominal aortic tissues and 7 AAA tissues. The GPL2507 platform was adopted (42). The GSE57691 dataset consisted of 10 control aortic tissues from organ donors, 20 aortic tissues from patients with small AAA (mean maximum aortic diameter of 54.3 ± 2.3 mm), and 29 aortic tissues from patients with large AAA (mean maximum aortic diameter of 68.4 ± 14.3 mm) (43). The GSE109639 dataset contained tissues from 6 sham mice and 6 mice with experimental AAA induced by CaCl_2_ (44). The GSE16676 dataset contained tissues from two healthy volunteers and four patients with AAA (18). A count matrix consisting of transcriptomics data of PBMCs from patients with AAA and healthy volunteers was deposited previously (17).

### Functional analysis

To explore the potential biological functions of the identified DEGs, the Database for Annotation, Visualization and Integrated Discovery^2^ was employed to conduct the GO analysis (45).

### ROC curve analysis

The ROC curve was generated using MedCalc software (version 19.0.7), and the AUC value with 95% confidence interval was calculated.

### Cell culture

VSMCs (American Type Culture Collection, Cat#PCS-100-012) were kindly provided by Dr. Tao Zhuang (Shanghai East Hospital, Tongji University School of Medicine) (46). VSMCs were maintained in Dulbecco’s Modified Eagle Medium (Gibco, US) supplemented with 10% fetal bovine serum (Gibco), 100 U/mL penicillin (Gibco), and 100 μg/mL streptomycin (Gibco). VSMCs were incubated at 37°C in a humidified atmosphere supplemented with 5% carbon dioxide. VSMCs at passages 12–15 were used in the present study. To build a reliable experimental AAA model *in vitro*, CaCl_2_ at different concentrations (0, 1, 10, and 100 mmol/L) was used to treat VSMCs for 24 h, as described previously (24, 26). Treatment with 100 mmol/L CaCl_2_ for 24 h was ultimately selected to induce AAA *in vitro*.

### Immunofluorescence analysis

For α-SMA immunofluorescence staining, the cells were fixed with 4% paraformaldehyde and blocked with phosphate-buffered saline containing 10% normal goat serum, as previously described (47). The cells were then incubated with primary antibody against α-SMA (Cat#ab7817, Abcam) followed by the corresponding secondary antibody. After, the cells were incubated with DAPI and images were captured using a microscope.

### qRT-PCR analysis

Total RNA was purified using TRIzol (Invitrogen, US), and cDNA libraries were synthesized with the Reverse Transcription Reagent kit (Takara, Japan). cDNA was submitted for qRT-PCR using SYBR Green mix (Takara) with GAPDH normalization. Primers (Supplementary Table 1) were purchased from Sangon Biological Engineering (Shanghai, China). The qRT-PCR data were analyzed using the comparative Ct (^Δ^ ^Δ^ Ct) method (48).

### Study participants

The study was performed in accordance with the Declaration of Helsinki and was approved by the ethics committee of our institution, and all patients gave written informed consent before operation (approval number: 2021-KY-079). Thoracic aortic tissues were collected from 11 patients with TAAD and eight control participants. Written informed consent was obtained from all of the study participants.

### Statistical analysis

The data are shown as the mean ± standard deviation. Paired groups were compared using the two-tailed Student’s *t-*test, while multiple groups were compared using the one-way analysis of variance with Tukey’s multiple comparisons test. A *p-*value of < 0.05 was considered statistically significant. Statistical analyses were performed using GraphPad Prism 7.

## Data availability statement

The datasets presented in this study can be found in online repositories. The names of the repository/repositories and accession number (s) can be found in the article/Supplementary material.

## Ethics statement

The studies involving human participants were reviewed and approved by The First Affiliated Hospital of USTC (approval number: 2021-KY-079). The patients/participants provided their written informed consent to participate in this study.

## Author contributions

XK and JG: conceptualization, supervision, project administration, and funding acquisition. PR and LG: methodology, software, and formal analysis. PR: validation, resources, and writing—original draft preparation. XK and PR: investigation. TC and HJ: data curation. XK: writing—review and editing. TC: visualization. All authors have read and agreed to the final version of the manuscript.

## Footnotes

1
http://www.ncbi.nlm.nih.gov/geo

2
https://david-d.ncifcrf.gov/
