# Supplementary material for: Identification of PTPN22 as a potential genetic biomarker for abdominal aortic aneurysm
Source: Front Cardiovasc Med. 2022 Dec 14;9:1061771. doi: 10.3389/fcvm.2022.1061771 (PMC9797128; doi:10.3389/fcvm.2022.1061771)
Supplement: Supplementary file 1 [file Data_Sheet_1.docx]

***Supplementary Material***


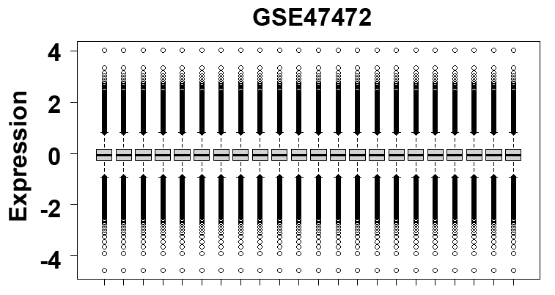


S**upplementary Figure 1.** Boxplot of the GSE47472 dataset after normalization.


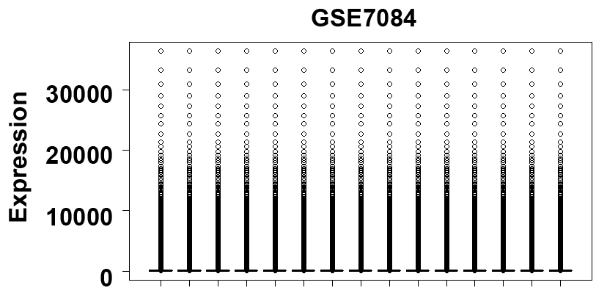


**Supplementary Figure 2.** Boxplot of the GSE7084 dataset after normalization.


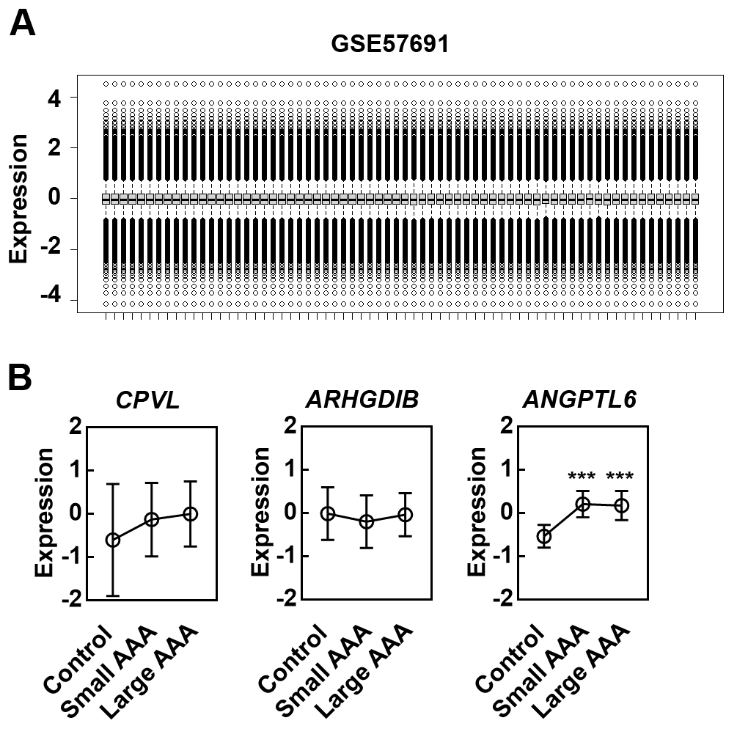


**Supplementary Figure 3.** Expression of identified genes in patients with abdominal aortic aneurysm (AAA). (A) Boxplot of the GSE57691 dataset after normalization. (B) Expression of *CPVL*, *ARHGDIB*, and *ANGPTL6* based on the GSE57691 dataset. The data are shown as the mean ± standard deviation of at least 10 patients. *, significantly different from control; ***, p < 0.001.


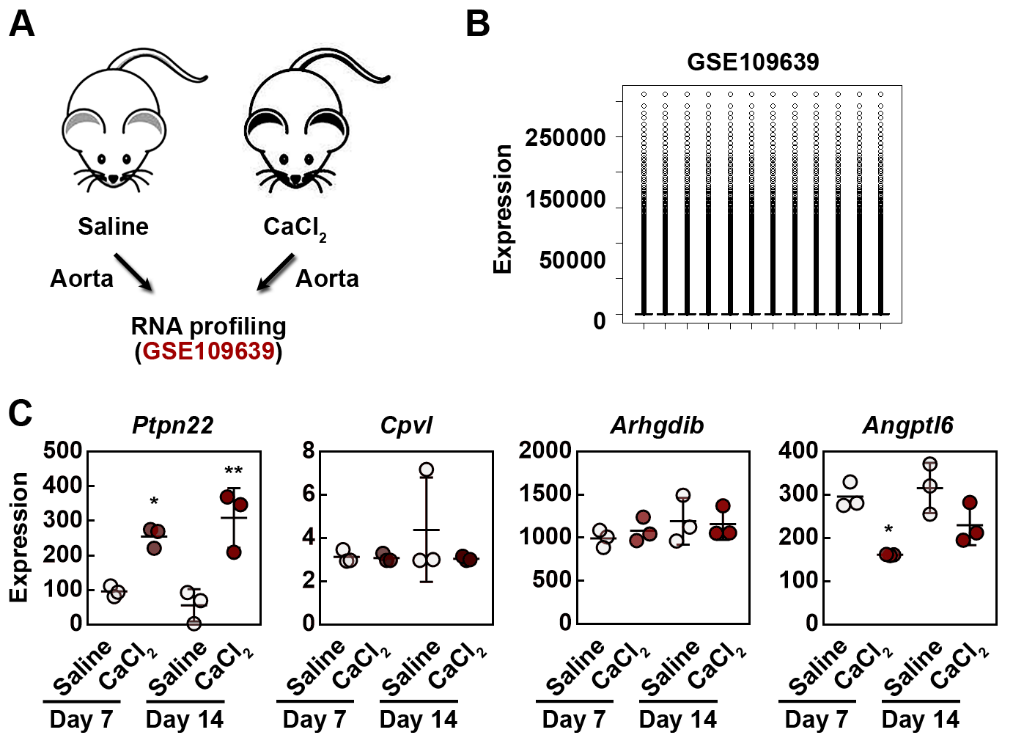


**Supplementary Figure 4.** Expression of identified genes in the experimental models. (A) Schematic of the GSE109639 dataset. (C) Boxplot of the GSE109639 dataset after normalization. (C) Expression of *PTPN22*, *CPVL*, *ARHGDIB*, and *ANGPTL6* in aortic tissues collected from sham mice and mice with abdominal aortic aneurysm (AAA) induced by calcium chloride (CaCl_2_). The data are shown as the mean ± standard deviation of three biological replicates. *, significantly different from saline group; *, p < 0.05; **, p < 0.01.


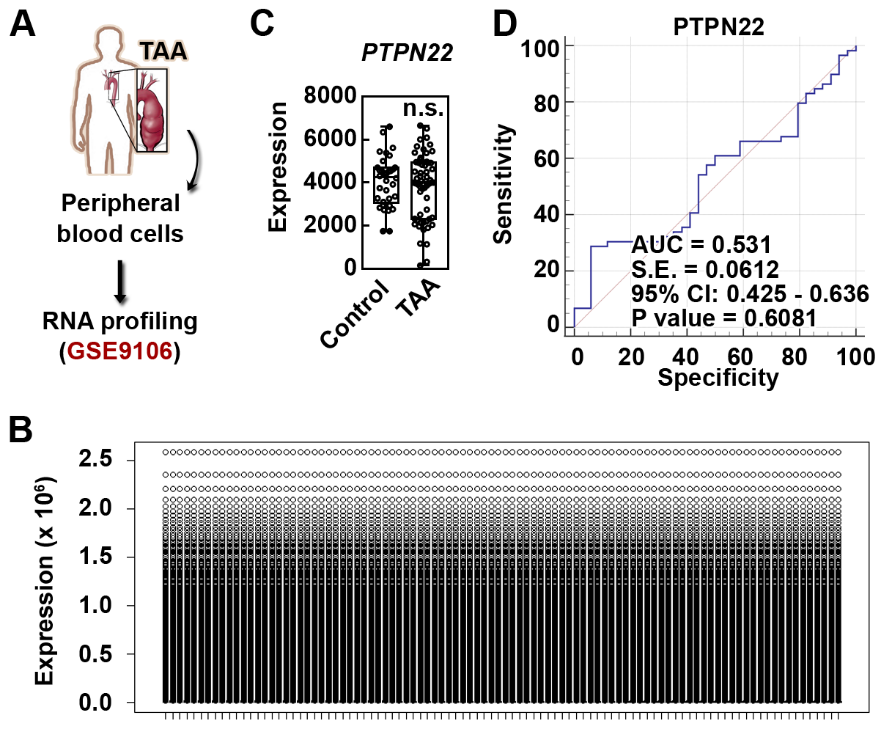


**Supplementary Figure 5.** *PTPN22* lacks diagnostic value for thoracic aortic aneurysm (TAA). (A) Schematic diagram of the GSE9106 dataset. Peripheral blood cells collected from control participants and patients with TAA were profiled by RNA sequencing. (B) Boxplot of the GSE9106 dataset after normalization. (C) Expression of *PTPN22* based on the GSE9106 dataset. The data are shown as the mean ± standard deviation of at least 34 patients. n.s., not significant. (D) Receiver operating characteristic curve analysis of *PTPN22* based on the GSE9106 dataset.


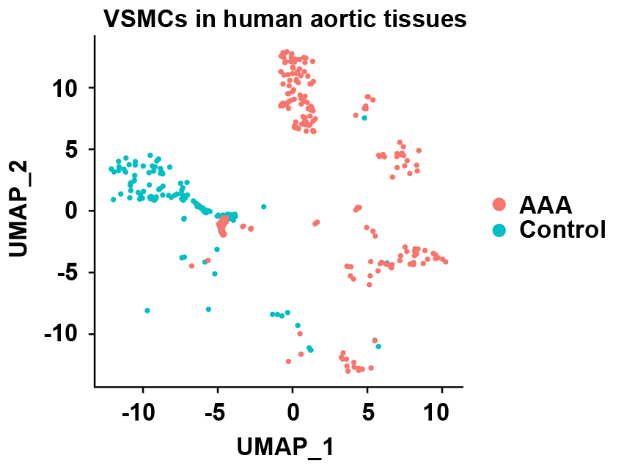


**Supplementary Figure 6.** UMAP plot showing vascular smooth muscle cells (VSMCs) in human aortic tissues from healthy control and patients with AAA.

**Supplementary Table 1.** Primers for qRT-PCR.

| Gene | Forward | Reverse |
| --- | --- | --- |
| *PTPN22* | GGCAATCCACCAAGTACAAGG | CCCTGGGTAGCAATATAAGCCT |
| *GAPDH* | AGGTCGGTGTGAACGGATTTG | GGGGTCGTTGATGGCAACA |
